# Gpr125 Marks Distinct Cochlear Cell Types and Is Dispensable for Cochlear Development and Hearing

**DOI:** 10.3389/fcell.2021.690955

**Published:** 2021-07-28

**Authors:** Haiying Sun, Tian Wang, Patrick J. Atkinson, Sara E. Billings, Wuxing Dong, Alan G. Cheng

**Affiliations:** ^1^Department of Otolaryngology-Head and Neck Surgery, Stanford University School of Medicine, Stanford, CA, United States; ^2^Department of Otorhinolaryngology, Union Hospital, Tongji Medical College, Huazhong University of Science and Technology, Wuhan, China

**Keywords:** Gpr125, cochlea, lesser epithelial ridge, hair cell, spiral ganglion neurons

## Abstract

The G protein-coupled receptor (GPR) family critically regulates development and homeostasis of multiple organs. As a member of the GPR adhesion family, Gpr125 (Adgra3) modulates Wnt/PCP signaling and convergent extension in developing zebrafish, but whether it is essential for cochlear development in mammals is unknown. Here, we examined the *Gpr125*^*lacZ/+*^ knock-in mice and show that Gpr125 is dynamically expressed in the developing and mature cochleae. From embryonic day (E) 15.5 to postnatal day (P) 30, Gpr125-β-Gal is consistently expressed in the lesser epithelial ridge and its presumed progenies, the supporting cell subtypes Claudius cells and Hensen’s cells. In contrast, Gpr125-β-Gal is expressed transiently in outer hair cells, epithelial cells in the lateral cochlear wall, interdental cells, and spiral ganglion neurons in the late embryonic and early postnatal cochlea. *In situ* hybridization for *Gpr125* mRNA confirmed *Gpr125* expression and validated loss of expression in *Gpr125^*lacZ/lacZ*^* cochleae. Lastly, *Gpr125*^*lacZ/+*^ and *Gpr125^*lacZ/lacZ*^* cochleae displayed no detectable loss or disorganization of either sensory or non-sensory cells in the embryonic and postnatal ages and exhibited normal auditory physiology. Together, our study reveals that Gpr125 is dynamically expressed in multiple cell types in the developing and mature cochlea and is dispensable for cochlear development and hearing.

## Introduction

G protein-coupled receptors (GPRs) form one of the largest gene families in the human genomes and serve critical functions across multiple organs (Pickering et al., 2008). Among the five subfamilies of mammalian GPRs, the adhesion family represents the second largest and consists of nine distinct subfamilies and 33 members, 10 of which have defined biological functions (Hamann et al., 2015; Vizurraga et al., 2020). For example, *Gpr56* deficiency causes brain malformation and myelination defects (Ganesh et al., 2020) and disrupts seminiferous tubule remodeling in the developing testis in mice (Chen et al., 2010). *Gpr124* knockout mice display abnormal angiogenesis in the developing forebrain and spinal cord, leading to hemorrhage and embryonic lethality (Cullen et al., 2011). Conditional deletion of *Gpr124* in adult mice disrupts the blood–brain barrier in ischemic conditions, underscoring its role in the mature organ (Chang et al., 2017). Another member of the GPR adhesion family, Gpr126, is required for myelination by Schwann cells in the mouse peripheral nerve system (Monk et al., 2011). Lastly, *Celsr1*-deficient mice demonstrate neural tube closure defects, abnormal skin hair patterning, and deformities (Curtin et al., 2003; Doudney and Stanier, 2005; Aw et al., 2016; Boucherie et al., 2018). These findings implicate significant roles for adhesion GPRs during development and homeostasis.

Several adhesion GPRs have been shown to be important for cochlear development. First, mutation of *Gpr98* causes moderate to severe congenital hearing loss in humans (Moteki et al., 2015; Bousfiha et al., 2017). In mice, *Gpr98* (or Very Large G-protein coupled receptor 1, Vlgr1) is required for the assembly of the ankle link complex and in the subsequent bundle development and survival of cochlear hair cells (McGee et al., 2006; Zou et al., 2015). As another adhesion GPR, Celsr1 is a planar cell polarity core protein expressed in cochlear and vestibular hair cells in mice (Curtin et al., 2003; Duncan et al., 2017). Its deficiency causes planar cell polarity defects of vestibular and cochlear hair cells and aberrant turning of axons in Type II spiral ganglion neurons (SGNs) (Curtin et al., 2003; Duncan et al., 2017; Ghimire et al., 2018). While these studies underscore the roles of adhesion GPRs in the inner ear, whether other adhesion GPRs also play similar roles is currently unknown.

As a member of the adhesion family, Gpr125 is a 57-kDa transmembrane signal transducer (Hamann et al., 2015; Wu et al., 2018). Gpr125 was originally described as a marker of spermatogonia stem cells (Seandel et al., 2007). More recently, Gpr125 has also been shown to be required for gastrulation and convergent extension movements by interacting with Disheveled proteins in zebrafish (Li et al., 2013). Here, we examined the *Gpr125*^*lacZ/+*^ reporter mice and show that Gpr125 is dynamically expressed in the embryonic and postnatal cochlea. We demonstrate that Gpr125-β-Gal is highly expressed in the LER and its derivatives in both the embryonic and postnatal cochleae. In addition, we found that Gpr125-β-Gal is transiently expressed in multiple other cell types in the late embryonic and early postnatal cochleae, including outer hair cells (OHCs), epithelial cells lining the lateral cochlear wall, interdental cells, and SGNs. Despite germline deletion of *Gpr125*, the embryonic and postnatal *Gpr125*^*lacZ/lacZ*^ cochleae show normal specification and organization of hair cell and supporting cell subtypes with no detectable convergent extension or hair cell polarity defects. The adult *Gpr125*^*lacZ/lacZ*^ mice also show normal auditory physiology. In summary, our study reveals that Gpr125 is dynamically expressed in multiple sensory and non-sensory cell types in the developing and mature cochlea, and is dispensable for the development and maintenance of the organ.

## Results

### Gpr125 Marks the Lesser Epithelial Ridge in the Early Embryonic Cochlea

In mice, the cochlear duct arises as a ventral out-pocketing of the developing otocyst around E11 (Driver et al., 2017). The prosensory region marked by Sox2 is flanked medially by the greater epithelial ridge and laterally by the lesser epithelial ridge (LER). At E15.5, prosensory cells are specified to become hair cells first in the mid-basal region, extending as a wave toward the apical turn over the next 2–3 days (Chen et al., 2002). Coinciding with this wave of cell specification, the cochlear duct lengthens with sensory and non-sensory cells precisely oriented, in processes called convergent extension and planar polarization.

**FIGURE 1:**
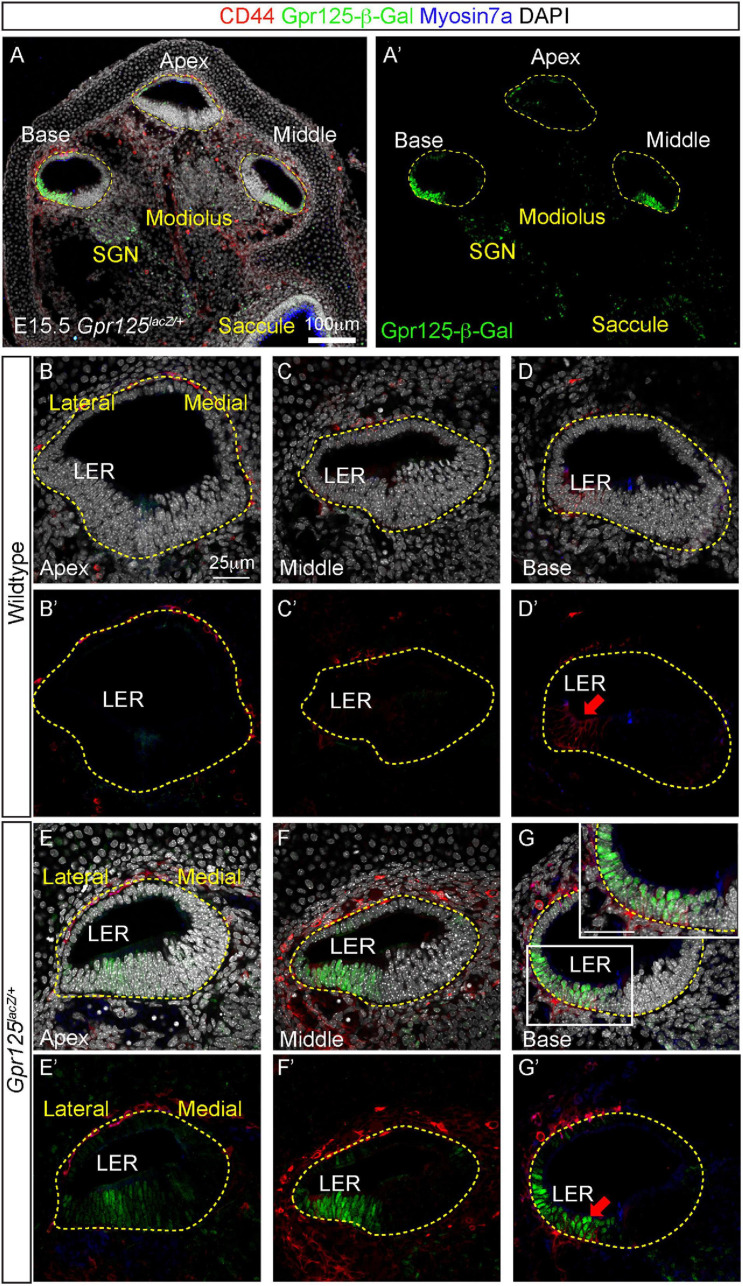
Expression patterns of Gpr125 in E15.5 *Gpr125*^*lacZ/+*^ mice. **(A,A’)** Low-magnification images of midmodiolar cochlear sections of E15.5 *Gpr125*^*lacZ/+*^ mice. Co-immunostaining of Gpr125-β-Gal (green), CD44 (red), and Myosin 7a (blue) shown in each cochlear turn. Gpr125-β-Gal-positive cells primarily occupied the floor throughout the entire cochlear duct. **(B–D’)** No Gpr125-β-Gal-positive cells were found in the wild-type cochleae. **(E–G’)** In the *Gpr125*^*lacZ/+*^ cochleae, Gpr125-β-Gal expression was detected in the floor and SGNs of each cochlear turn. Expression is spatially restricted to the LER and outer sulcus, and more robust in the middle and base turn relative to the apex. Gpr125-β-Gal signal in the outer sulcus extends to the lower half of the lateral cochlear wall. Gpr125-β-Gal expression overlapped with CD44, which marks the LER only in the basal turn at this age. Inset in panel **(G)** shows high-magnification image. CD44 is also occasionally expressed in the periotic mesenchyme surrounding the cochlear duct. Red arrow marks CD44-positive cells in panels **(D’,G’)**. *n* = 4 for wild type, *n* = 3 for *Gpr125*^*lacZ/+*^.

In the embryonic (E) 15.5 cochlea, prosensory cells are specified to become hair cells (Driver and Kelley, 2020). The prosensory domain resides in the floor of the cochlear duct between the greater and lesser epithelial ridges. Hair cell specification first occurs in the basal turn and then extends in a wave toward the apex (Chen et al., 2002). To study the expression pattern of *Gpr125* at this developmental stage, we examined the *Gpr125*^*lacZ/+*^ knock-in mouse, in which a lacZ-neomycin cassette was inserted into exon 15 (see *Materials and Methods* for details). The cochleae were immunostained for lacZ [β-galactoside (β-Gal)], Myosin7a, and CD44 (Figure 1A). At E15.5, Myosin7a marks outer and inner hair cells, and CD44 marks the LER only in the basal turn and occasionally expressed in the periotic mesenchyme surrounding the cochlear duct (Figures 1A,A’; Hasson et al., 1995; Hertzano et al., 2010). As controls, no Gpr125-β-Gal-positive cells were observed in wild-type cochleae (Figures 1B–D). In each turn of *Gpr125*^*lacZ/+*^ cochleae, robust nuclear Gpr125-β-Gal expression was detected in the LER and outer sulcus in the lateral cochlear ductal floor and in the SGNs in the modiolus (Figures 1E–G, Supplementary Figures 1A–D). Expression in the apical turn is noticeably less intense than the middle and basal turns in both the cochlear duct and SGNs, suggesting an increasing apical–basal gradient. In the basal turn where specification of Myosin7a^+^ hair cells has occurred, we found CD44 expression overlapping with β-Gal expression in the LER (Figure 1G). We also observed a relatively weaker Gpr125-β-Gal signal in the outer sulcus extending to the lower half of the lateral cochlear wall (Figures 1E–G). Taken together, these results indicate that Gpr125 is expressed in the LER, preceding the onset of CD44 expression and sensory cell specification in the early embryonic cochlea.

### Gpr125 Expression Broadens in the Late Embryonic Cochlea

**FIGURE 2:**
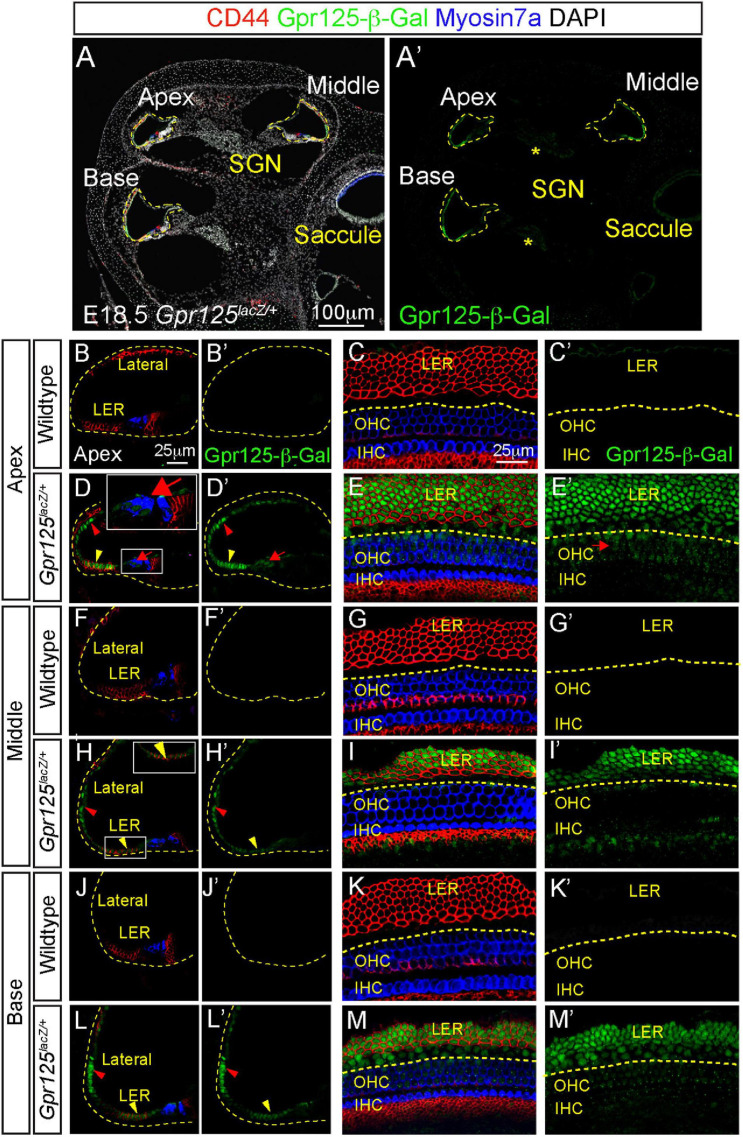
Expression patterns of Gpr125 in E18.5 *Gpr125*^*lacZ/+*^ mice. **(A,A’)** Representative images of midmodiolar cochlear sections of E18.5 *Gpr125*^*lacZ/+*^ mice immunostained for Gpr125-β-Gal (green), CD44 (red), and Myosin7a (blue). Gpr125-β-Gal expression located in the LER, lateral cochlear wall, OHCs and SGNs (asterisks). **(B–C’)** CD44 marks the LER and inner phalangeal cells inside the cochlear duct and also the mesenchymal cells outside the roof. No Gpr125-β-Gal expression was observed in the wild-type cochleae. **(D,D’)** Gpr125-β-Gal expression was detected at the LER (yellow arrowhead) extending to the lateral cochlear wall (red arrowhead) at the apical turn of *Gpr125*^*lacZ/+*^ cochlea. Low expression was also noted in outer hair cells (red arrows). **(E,E’)** Confocal images of whole mount cochlea (apical turn shown) from E18.5 *Gpr125*^*lacZ/+*^ mice showing co-expression of Gpr125-β-Gal and CD44 in the LER. Gpr125-β-Gal expression was also detected in the CD44-negative Hensen’s cells and OHCs (red arrows). **(F–G’)** No Gpr125-β-Gal signal was detected at the middle turns of the wild-type cochlea. **(H–I’)** Like the apical turn, Gpr125-β-Gal was detected in the lateral cochlear wall and LER in the middle turn of *Gpr125*^*lacZ/+*^ cochlea (red and yellow arrowheads, respectively). Gpr125-β-Gal was not detected in hair cells. **(J–K’)** No Gpr125-β-Gal signal was detected at the base of the wild-type cochlea. **(L–M’)** Gpr125-β-Gal is expressed in the LER and lateral cochlear wall in the base turn of *Gpr125*^*lacZ/+*^ cochlea. High-magnification images shown in inset for panels **(D,H)**. GER, greater epithelial ridge; LER, lesser epithelial ridge; IHC, inner hair cells; OHC, outer hair cells; SGN, spiral ganglion neurons.

At E18.5, both outer and inner hair cells and most support cell subtypes are specified in all three cochlear turns (Kolla et al., 2020). In the *Gpr125*^*lacZ/+*^ cochlea, strong β-Gal signal was detected in the LER, lateral cochlear wall, and weak signal in the modiolus (Figure 2A). No Gpr125-β-Gal expression was detected in the wild-type cochlea (Figures 2B,C,F,G,J,K). In the lateral cochlear wall, Gpr125-β-Gal is strongly expressed in cells spanning from the LER to the lateral cochlear wall (Figures 2D,H,L). This expression is broader and more intense than that of E15.5, when Gpr125-β-Gal expression is restricted to the lower half of the lateral wall. Within the LER domain, cells located in the lateral two-thirds strongly express Gpr125-β-Gal, whereas the two rows of cells residing in the medial portion show weaker but detectable expression in all three cochlear turns (Figures 2D,E,H,I,L,M). We immunostained for CD44 and found that CD44 marks the LER, inner phalangeal cells inside the cochlear duct, and also the mesenchymal cells outside the roof (Figures 2B–D’), and overlapped with Gpr125-β-Gal expression in the LER (Figures 2D,H,L). Medial LER cells, which presumably give rise to Hensen’s cells, lack CD44 expression (Figures 2D,H,L). No apical–basal gradient was

observed with Gpr125-β-Gal expression in the LER at this age (Figures 2D,E,H,I,L,M). On the other hand, Gpr125-β-Gal is weakly expressed among OHCs only in the apical turn (Figures 2D,E) at this time point. As hair cells are more mature in the basal turn, these data suggest that Gpr125 is transiently expressed in OHCs and is rapidly downregulated as the hair cells mature. Relative to E15.5, Gpr125 expression at E18.5 is less restricted, labeling the LER, lateral cochlear wall, and modiolus.

### Expression Pattern of Gpr125 in the Postnatal Cochlea

**FIGURE 3:**
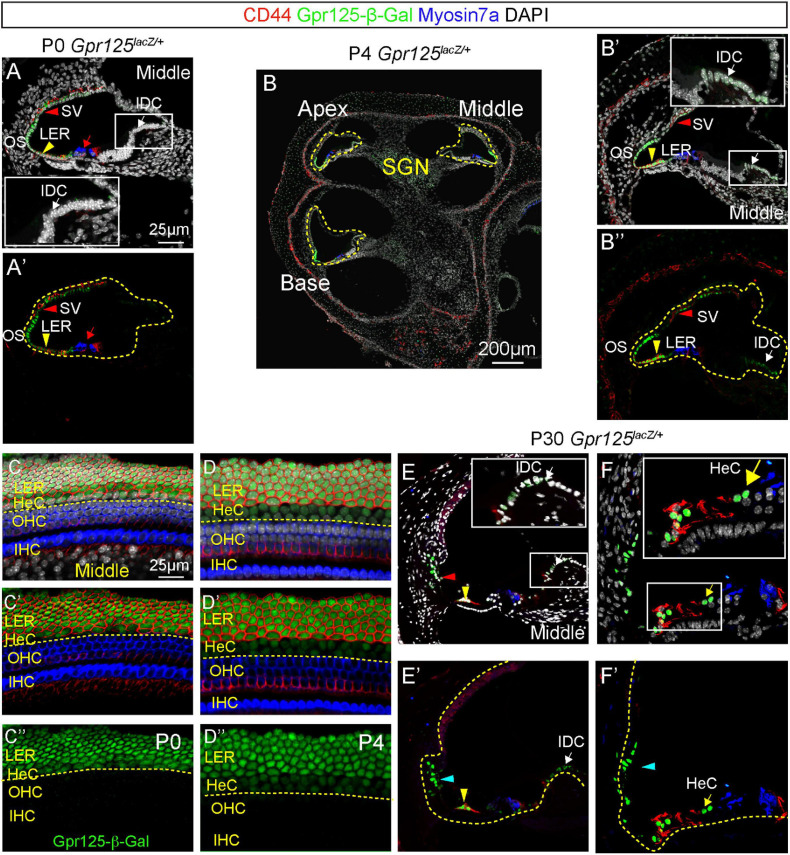
Expression of Gpr125 in the postnatal cochlea. **(A,A’)** Representative image of section of the middle turn of P0 *Gpr125*^*lacZ/+*^ cochlea. Gpr125-β-Gal was detected in the lateral cochlear wall, stria vascularis (SV, red arrowheads), outer sulcus, SGNs, and LER (yellow arrowheads). CD44 marks Claudius cells. Inset demonstrates high magnification of interdental cells (IDC, white arrows) in panel **(A)**. No expression was detected in the IDCs or outer hair cells (red arrows). **(B)** Midmodiolar sections of P4 *Gpr125*^*lacZ/+*^ cochlea showing Gpr125-β-Gal expression in the LER, SGNs, SV, and outer sulcus. **(B’,B”)** At P4, Gpr125-β-Gal expression was detected at the lateral cochlear wall, SV (red arrowheads), outer sulcus, IDCs (white arrows), and LER (yellow arrowheads). **(C–D”)** Whole mount preparation of P0 and P4 *Gpr125*^*lacZ/+*^ cochleae showing β-Gal and CD44 expression in LER, and Gpr125-β-Gal expression alone in Hensen’s cells (HeC). **(E–F’)** At P30, Gpr125-β-Gal expression was detected in Claudius cells (yellow arrowheads), Hensen’s cells (yellow arrows), and outer sulcus (cyan arrowheads). Relative to P4, Gpr125-β-Gal expression in the IDC (white arrows) is more intense. Insets show magnification of boxed areas in A–E. SV, stria vascularis; LER, lesser epithelial ridge; OHC, outer hair cells; SGN, spiral ganglion neurons; IHC, inner hair cells.

The postnatal cochlea undergoes several morphological changes, including opening of the tunnel of Corti around P5–P7 and the apoptosis of the GER between P7 and P10 (Peeters et al., 2015; Basch et al., 2016). To determine the expression of Gpr125 in the postnatal cochlea, we immunolabeled Gpr125-β-Gal in the *Gpr125*^*lacZ/+*^ cochlea at P0, P4, and P30. We first analyzed the wild-type cochlea at P0, P4, and P30 and no Gpr125-β-Gal-positive cells were detected (not shown). Similar to E18.5, Gpr125-β-Gal-positive cells were primarily observed in the LER and lateral cochlear wall in the P0 *Gpr125*^*lacZ/+*^ cochlea (Figure 3A). In contrast to E18.5, the Gpr125-β-Gal signal is absent in the OHCs in all turns at P0 (Figures 3A,C), supporting the observation that Gpr125 is transiently expressed in the embryonic OHCs. Relative to E18.5, Gpr125-β-Gal expression is more robust in the CD44-positive lateral LER (presumed Claudius cells) and outer sulcus (Figure 3C). At both P0 and P4, expression of Gpr125-β-Gal is more intense in the medial, CD44-negative LER (presumed Hensen’s cells) than at E18.5. Moreover, the expression of β-Gal in the outer sulcus and LER is more intense at P4 compared to P0 (Figures 3B,D). In the lateral cochlear wall, Gpr125-β-Gal signal was detected in the stria vascularis, with signal appearing the highest in the epithelial layer at both P0 and P4 (Figures 3A,B). Compared to P0, Gpr125-β-Gal expression in the lateral wall is markedly lower at P4 (Figure 3B). Lastly, we detected Gpr125-β-Gal signal in interdental cells in P4 but not P0 *Gpr125*^*lacZ/+*^ cochleae (Figures 3A,B).

In the mature, P30 cochlea, Gpr125-β-Gal expression is still robust in the outer sulcus, Claudius cells, and Hensen’s cells. However, Gpr125-β-Gal signal is no longer detectable in the stria vascularis (Figures 3E,F). Furthermore, relative to P4, β-Gal expression in the interdental cells appears more intense at P30. There is no difference in the immunolabeling of β-Gal from the apical to basal turns (data not shown). Together, these data indicate that Gpr125 is dynamically expressed in multiple cell types in the postnatal cochlea, except in Claudius and Hensen’s cells where expression is consistent.

### Cochlear Development in the *Gpr125*^*lacZ/lacZ*^ Mice

**FIGURE 4:**
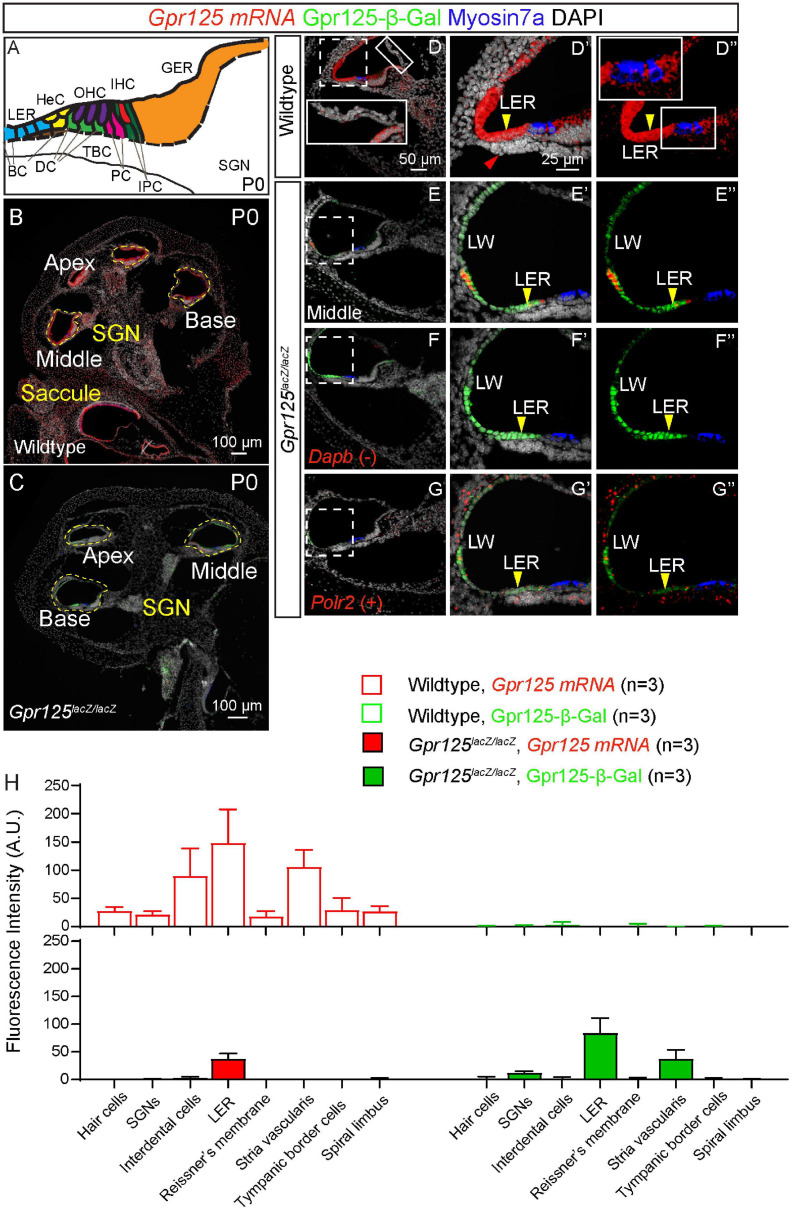
*Gpr125* mRNA expression in wild-type and *Gpr125*^*lacZ/lacZ*^ cochleae. **(A)** Schematic of P0 cochlea showing subtypes of hair cells and supporting cells. BC, Boettcher cells; DC, Deiters’ cells; TBC, tympanic border cells; PC, pillar cells; IPC, inner phalangeal cells; LER, lesser epithelial ridge; HeC, Hensen’s cells; OHC, outer hair cells; IHC, inner hair cells; GER, greater epithelial ridge; SGNs, spiral ganglion neurons. **(B,C)** Low-magnification image of cryosection demonstrates robust *Gpr125 mRNA* expression in P0 wild-type cochlea. *Gpr125 mRNA* expression is low or undetectable in most regions in the *Gpr125*^*lacZ/lacZ*^ cochlea, with the exception of the lateral wall and LER where significant expression remained detectable. **(D–D”)** High-magnification images of cochlear section showing robust *Gpr125 mRNA* signals in the wild-type cochlea. Robust *Gpr125 mRNA* signals were detected at the lateral cochlear wall (LW), outer sulcus, and lesser epithelial ridge, and at lower levels in the organ of Corti, Reissner’s membrane, tympanic border cells (red arrow), spiral limbus, and greater epithelial ridge. No Gpr125-β-Gal-positive cells were detected in the wild-type cochlea. **(E–E”)** Relative to the wild-type cochlea, *Gpr125 mRNA* signal is dramatically lower in the *Gpr125*^*lacZ/lacZ*^ cochlea. **(F–F”)** Labeling for Dihydrodipicolinate reductase (*Dapb*) is used as a negative control. **(G–G”)** Labeling for RNA polymerase II (*Polr2*) is used as a positive control. **(H)** Fluorescence intensity of *Gpr125 mRNA* and Gpr125-β-Gal protein in cell types of interest. *Gpr125* mRNA signal is the highest in the LER in wild-type cochleae. Similarly, immunolabeling for β-Gal protein expression is the strongest in the LER in *Gpr125*^*lacZ/lacZ*^ cochleae. The fluorescence of *Gpr125* mRNA correlated to β-Gal (*R*^2^ = 0.82, *p* < 0.01, Pearson’s correlation). Data are presented as mean ± SD.

To validate Gpr125 deletion, *in situ* hybridization using probes specific for the Gpr125 exons 15–19 was performed in P0 wild-type and *Gpr125*^*lacZ/lacZ*^ mice. As the lacZ cassette is inserted into exon 15, mRNA expression detected by these probes was expected to be lower in *Gpr125*^*lacZ/lacZ*^ mice. After combining immunostaining for β-Gal and Myosin7a with *in situ* hybridization, we observed abundant *Gpr125* mRNA expression in multiple cochlear regions in wild-type mice and a notable absence of *Gpr125* transcripts in the same region in *Gpr125*^*lacZ/lacZ*^ cochleae (Figures 4A–C). *Gpr125* mRNA signal is evident in several areas of the P0 wild-type cochleae, including hair cells, interdental cells, LER, Reissner’s membrane, stria vascularis, tympanic border cells, spiral limbus, and the modiolus (presumed spiral ganglia neurons, SGNs) (Figures 4B,D). This pattern is noticeably broader than that of Gpr125-β-Gal. For example, *Gpr125 mRNA* was detected in P0 interdental cells and stria vascularis where no β-Gal signal was detected. No β-Gal signal was observed in the wild-type cochleae. In P0 *Gpr125*^*lacZ/lacZ*^ cochleae, β-Gal signal was mainly noted in SGNs, LER, and stria vascularis similar to *Gpr125*^*lacZ/+*^ cochleae (Figures 4C,E). Compared to wild-type cochlea, markedly lower *Gpr125* mRNA signal was detected in the stria vascularis and LER in the *Gpr125*^*lacZ/lacZ*^ cochleae (Figure 4E), indicating that *Gpr125* transcripts are markedly reduced in the homozygous cochleae. The specificity of the signal was confirmed by the lack of signal in negative controls (using probes against *Dapb*) (Figure 4F). The signal intensity of each region was compared to positive controls (using probes against *Polr2*), which displayed robust staining (Figure 4G). Because some mRNA signal remained in the *Gpr125*^*lacZ/lacZ*^ cochleae, we quantified the levels of β-Gal and *Gpr125* mRNA signal in defined regions of the cochlea. The *Gpr125* mRNA signal is the most intense in the LER and stria vascularis in the wild-type cochleae. Similarly, β-Gal expression in these two regions is the most intense in the *Gpr125*^*lacZ/lacZ*^ cochleae (Figure 4H). Relatively lower *Gpr125* mRNA expression was detected in hair cells, interdental cells, Reissner’s membrane, tympanic border cells, and spiral limbus of wild-type cochleae, whereas no *Gpr125* mRNA signal was detected in those regions in *Gpr125*^*lacZ/lacZ*^ cochlea, suggesting that *Gpr125* is absent in these regions. Overall, *Gpr125* mRNA levels significantly correlated with the β-Gal signals (*R*^2^ = 0.82, *p* < 0.01, Pearson’s correlation, Figure 4H). Together, these data validate the *Gpr125*^*lacZ/lacZ*^ cochlea as a model to assess *Gpr125* deficiency.

### Dynamic Expression of Gpr125 in SGNs

**FIGURE 5:**
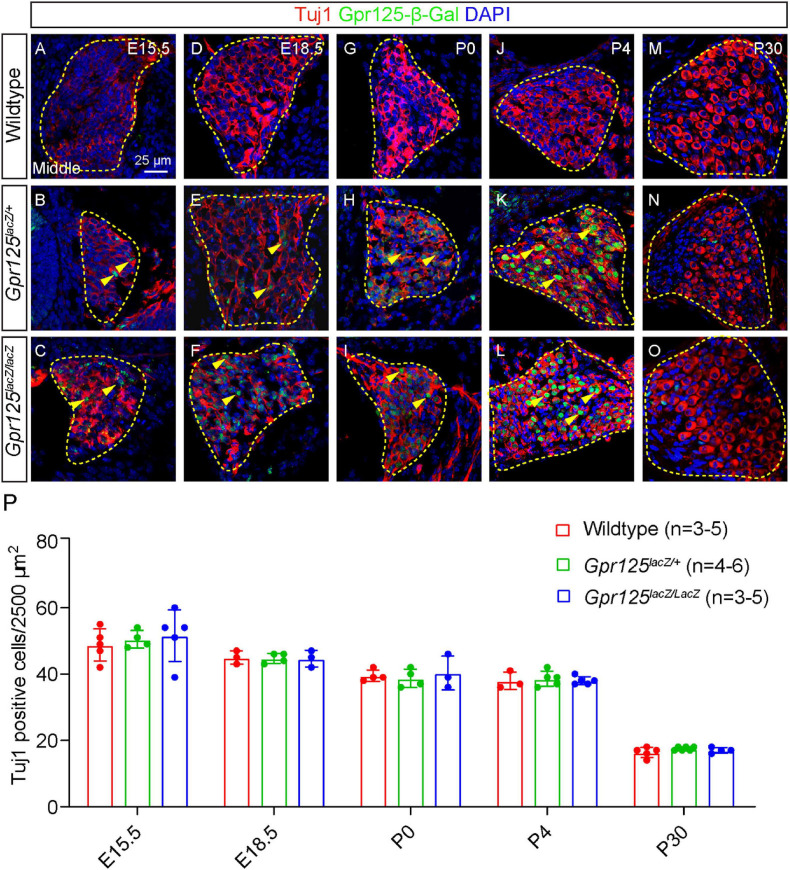
Gpr125 deficiency does not impair spiral ganglion neuron development. Representative sections of Rosenthal’s canal from the middle turn of wild-type, *Gpr125*^*lacZ/+*^, and*Gpr125^*lacZ/lacZ*^* cochleae. All sections were co-stained for β-Gal (green), Tuj1 (red), and DAPI (blue). **(A–I)** Between E15.5 and P0, Gpr125-β-Gal expression is detected in a subset of Tuj1-positive SGNs in the *Gpr125*^*lacZ/+*^ and *Gpr125^*lacZ/lacZ*^* cochleae (yellow arrowheads). **(J–L)** At P4, β-Gal expression is notably more intense in most Tuj1-positive SGNs. **(M–O)** At P30, Gpr125-β-Gal was undetectable in Tuj1-positive SGNs, but was noted in a few surrounding Tuj1-negative cells. Gpr125-β-Gal-positive signal was not observed in wild-type SGNs across ages. **(P)** Quantification of Tuj1-positive neurons showing no significant difference in counts among wild-type, *Gpr125*^*lacZ/+*^, and *Gpr125*^*lacZ/lacZ*^ cochleae in all ages examined (*p* > 0.05, one-way ANOVA). Data are presented as mean ± SD.

We next characterized Gpr125 expression in the SGNs in the embryonic and postnatal cochlea. At E15.5 and E18.5, a relatively low expression of Gpr125-β-Gal was detected in Tuj1^+^ (class III beta-tubulin) SGNs in *Gpr125^*lacZ/+*^* and *Gpr125*^*lacZ/lacZ*^ cochleae (Figures 5A–F). In the modiolus, β-Gal expression is limited to SGNs in the modiolus at E15.5, E18.5, and P0. Relative to these ages, β-Gal expression is noticeably higher at P4 (Figures 5G–L). By P30, we could not detect any Gpr125-β-Gal signal in Tuj1-positive SGNs, while some Gpr125-β-Gal-positive, Tuj1-negative cells (presumably glial or satellite cells) were observed (Figures 5M–O). No apical-to-basal gradient of Gpr125-β-Gal expression was observed except for E15.5 (Supplementary Figure 1). Taken together, these findings demonstrate that Gpr125 expression in SGNs increases from embryonic to early postnatal ages, before becoming undetectable in the mature cochlea.

To investigate whether Gpr125 is required for SGN development and survival, we quantified the Tuj1-positive cells in middle turns of wild-type, *Gpr125*^*lacZ/+*^, and *Gpr125*^*lacZ/lacZ*^ cochleae. No significant differences were observed in the density of Tuj1-positive SGNs among all three groups (Figure 5P). Our results indicate that Gpr125 is not required for SGN development or survival in the embryonic, neonatal, or adult cochlea.

### Normal Cochlear Development in the *Gpr125*^*lacZ/lacZ*^ Mice

**FIGURE 6:**
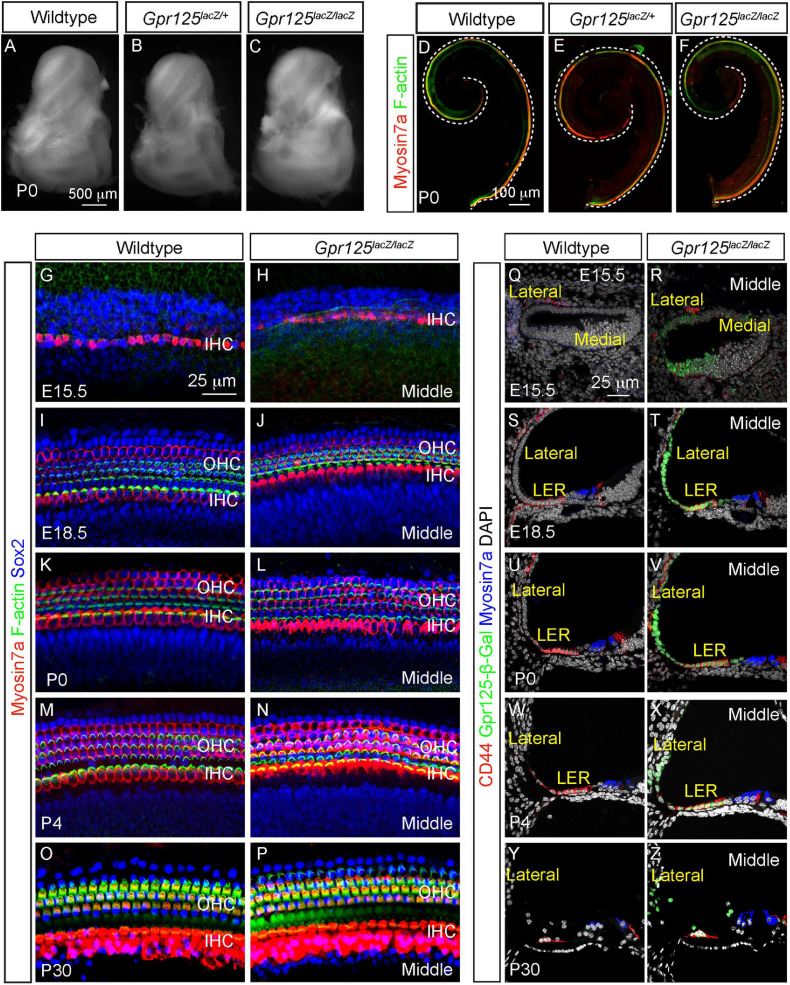
Gpr125 is dispensable for cochlear development. **(A–C)** Otic capsule from P0 wild-type, *Gpr125*^*lacZ/+*^, and *Gpr125*^*lacZ/lacZ*^ mice. **(D–F)** Whole mount preparation of cochleae from the wild-type, *Gpr125*^*lacZ/+*^, and *Gpr125*^*lacZ/lacZ*^ mice showing no differences in length. **(G–P)** Whole mount preparation of E15.5, E18.5, P0, P4, and P30 cochleae immunostained for Myosin7a (red), F-actin (green), and Sox2 (blue), demonstrating no detectable loss of hair cells, hair bundles, or supporting cells in the *Gpr125*^*lacZ/lacZ*^ cochleae. Images were taken from the middle turn. **(Q–Z)** Representative sections through the middle turn of E15.5, E18.5, P0, P4, and P30 cochleae from wild-type and *Gpr125*^*lacZ/lacZ*^ mice. *Gpr125*^*lacZ/lacZ*^ mutants demonstrate relatively normal cochlear morphology, including in the stria vascularis and LER that strongly express Gpr125-β-Gal.

Gpr125 has been shown to modulate Wnt/PCP signaling and to be required for gastrulation in zebrafish (Li et al., 2013). Shortened cochlea as a result of defective convergent extension is a hallmark of PCP defects (Driver et al., 2017; Najarro et al., 2020). To test whether convergent extension was perturbed by *Gpr125* deficiency, we examined the otic capsule from P0 wild-type, *Gpr125*^*lacZ/+*^, and *Gpr125*^*lacZ/lacZ*^ mice and found them to be morphologically indistinguishable (Figures 6A–C). Moreover, length of the *Gpr125*^*lacZ/lacZ*^ cochleae was comparable to those of wild-type and *Gpr125*^*lacZ/+*^ littermates (Figures 6D–F, Supplementary Figure 2A), suggesting no obvious convergent extension defects. By immunostaining hair cells, bundles, and supporting cells, we found no hair cell or supporting cell loss or disorganization in the *Gpr125*^*lacZ/lacZ*^ cochlea at any ages examined (Figures 6G–P, Supplementary Figures 2B–N). Phalloidin staining showed that stereociliary bundles are grossly intact in all ages of *Gpr125*^*lacZ/lacZ*^ mice (Figures 6G–P and Supplementary Figures 2B–K). We also examined the stria vascularis, the lateral cochlear wall, and LER, where Gpr125 is robustly expressed, and found no cell loss or morphologic anomalies between E15.5 and P30 (Figures 6Q–Z). Collectively, these results suggest that Gpr125 is dispensable for cochlear development including specification and polarization of hair cells.

### *Gpr125*^*lacZ/lacZ*^ Mice Show No Hearing Loss

**FIGURE 7:**
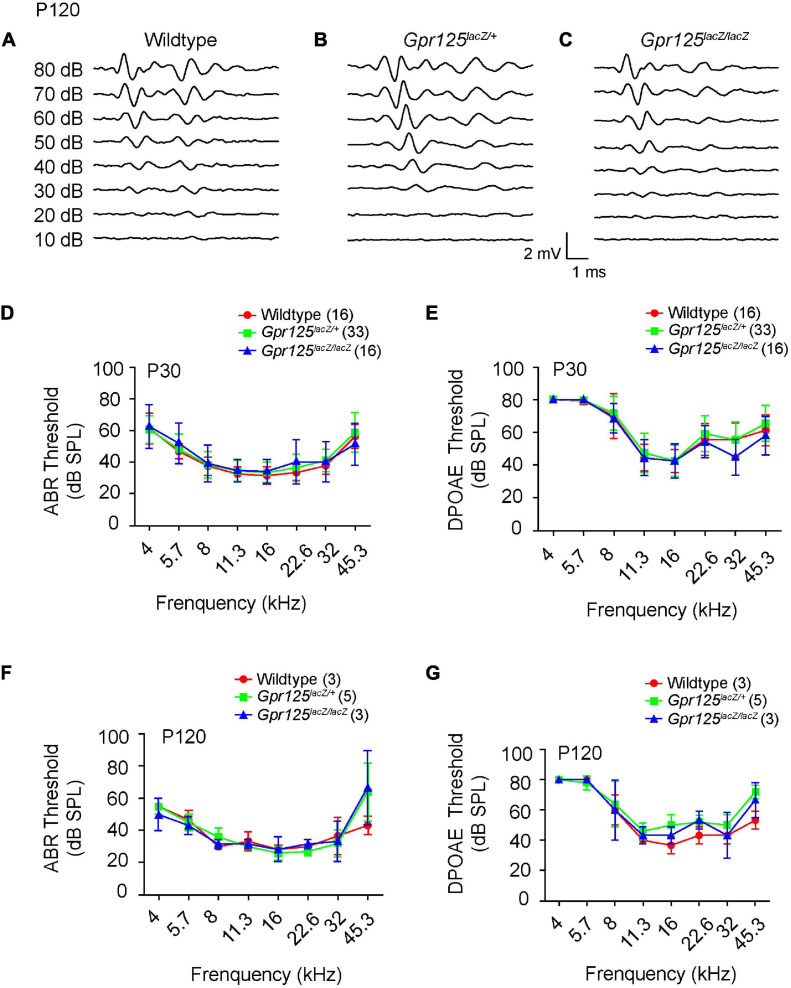
*Gpr125*^*lacZ/lacZ*^ mice exhibit normal auditory physiology. **(A–C)** Representative ABR waveforms of P120 wild-type, *Gpr125*^*lacZ/+*^, and *Gpr125*^*lacZ/lacZ*^ mice. **(D–G)** Comparable ABR and DPOAE thresholds of P30 and P120 wild-type, *Gpr125*^*lacZ/+*^, and *Gpr125*^*lacZ/lacZ*^ littermates. No significant differences were observed among wild-type, *Gpr125*^*lacZ/+*^, and *Gpr125*^*lacZ/lacZ*^ mice (*p* > 0.05, one-way ANOVA). Data are presented as mean ± SD.

To explore whether Gpr125 is required for auditory function, ABR thresholds were examined from P30 and P120 *Gpr125*^*lacZ/+*^, *Gpr125*^*lacZ/lacZ*^, and wild-type littermate control mice (Figures 7A–G). ABR thresholds (4–45.3 kHz) showed no significant differences among three genotypes tested at P30 or P120 (*p* > 0.05, one-way ANOVA) (Figures 7D,F). We also measured the DPOAE responses of P30 and P120 *Gpr125*^*lacZ/lacZ*^ mice and found no differences in thresholds compared with wild-type and *Gpr125*^*lacZ/+*^ mice (Figures 7E,G). Together these results indicate Gpr125 is not required for auditory function in mice.

## Discussion

**FIGURE 8:**
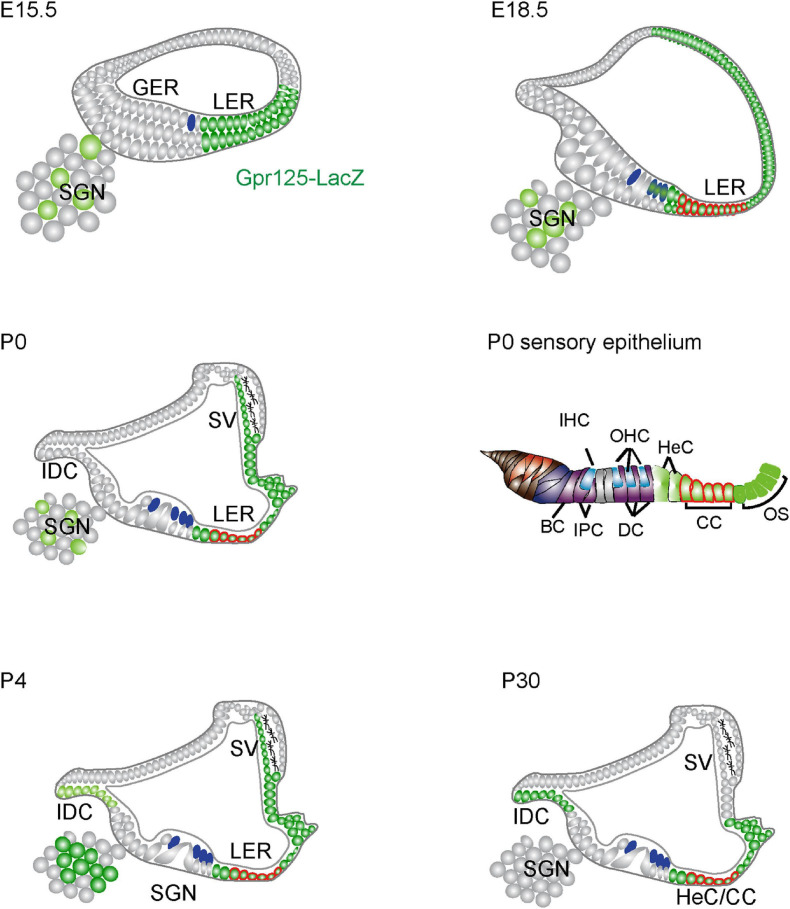
Schematic depiction of Gpr125 expression in the developing and mature cochlea. At E15.5, Gpr125 is expressed in the LER, LW, and SGNs. At E18.5, Gpr125 is expressed broadly and strongly in the LER and lateral cochlear wall. It is expressed at lower levels in the OHCs and SGNs. At P0 and P4, Gpr125 is expressed in the LER, SV, and SGNs. The IDCs do not express Gpr125 at P0 and display robust expression later at P4 and P30. At P30, Gpr125 is expressed in the Claudius cells, Hensen’s cells, and outer sulcus, but is no longer expressed in the SV and SGNs. SGN, spiral ganglion neurons; GER, greater epithelial ridge; LER, lesser epithelial ridge; SV, stria vascularis; OHC, outer hair cells; IHC, inner hair cells; HC, Hensen’s cell, IDC, interdental cell; IPC, inner phalangeal cells; PC, pillar cells; DC, Deiters’ cells; CC, Claudius’s cells; OS, outer sulcus cells; BC, border cells.

In this study, we systematically characterized the expression pattern and the role of Gpr125 during the cochlea development and maturation by employing the *Gpr125*^*lacZ/+*^ knock-in mouse line. We found Gpr125 to be dynamically expressed in multiple cell types in the embryonic and postnatal cochlea, spanning the lateral cochlear wall, LER, organ of Corti, interdental cells, and modiolus (Figure 8). Gpr125 consistently marks the LER and its derivatives, Claudius and Hensen’s cells, throughout the developmental stages examined. Lastly, *Gpr125*^*lacZ/lacZ*^ mice display normal cochlear development and auditory function, suggesting that Gpr125 is dispensable for cochlear development and maintenance.

### Markers of the Lesser Epithelial Ridge and Derivatives

The embryonic and postnatal cochlea is radially patterned in a manner perpendicular to the tonotopic gradient arranged longitudinally along the cochlea. By E11.0, the cochlear duct has already developed into five distinct structures: the prospective LER, the Reissner’s membrane, the greater epithelial ridge (also known as Kölliker’s organ), the stria vascularis, and the prosensory domain (Driver and Kelley, 2020). BMP4 marks the LER between E16 and P1 (Morsli et al., 1998), whereas CD44 marks the lateral LER in embryonic and neonatal cochlea (Hertzano et al., 2010). Unlike CD44, Gpr125 expression spans the lateral and medial LER and its derivatives in the embryonic, neonatal, and adult cochlea, consistent with recently published single-cell RNA-sequencing data (Kolla et al., 2020). The differential expression of CD44 and Gpr125 suggests that there are at least two distinct groups of LER cells, which likely give rise to Hensen’s cells and Claudius cells in adult cochlea. Therefore, like the organ of Corti and GER, the LER is also radially patterned as the cochlea matures (Jansson et al., 2019; Munnamalai and Fekete, 2020).

While Hensen’s cells and Claudius cells can be distinguished using molecular markers and spatially, whether they serve distinct functions in the cochlea is not known. A recent study characterized the requirement of the Notch ligand Jagged1 for the formation of Hensen’s cells in the embryonic cochlea (Chrysostomou et al., 2020). Interestingly, LER cells formed Claudius cells instead of Hensen’s cells in the absence of Jagged1. The use of Gpr125 as a marker can further facilitate studies of radial patterning of the LER and the functions of distinct cell populations therein. It is important, however, to note that the β-Gal signal in the P0 *Gpr125-LacZ* cochleae is noticeably less broad and intense than the *Gpr125 mRNA* signal in wild-type animals.

### Gpr125 Is Dispensable for Cochlear Development and Function

Gpr125 has been shown to be required for gastrulation during development of zebrafish (Li et al., 2013). Given its role as a modulator of the Wnt/PCP signaling, we hypothesized that *Gpr125* deficiency would perturb the development of the mouse cochlea. To our surprise, Gpr125 is dispensable for the cell survival, specification, and organization in all cochlear regions where it is expressed. More specifically, cochlear length is unaffected and organization of hair cells and supporting cells appear normal, suggesting no PCP defects.

In the P30 cochlea when the auditory system is functionally mature, we found that *Gpr125* deletion does not lead to changes in thresholds of ABR and DPOAE. The endocochlear potential established by the stria vascularis, which was shown to express Gpr125-β-Gal at several developmental stages, is required for hair cell function. Since we did not detect any ABR/DPOAE changes in *Gpr125*^*lacZ/lacZ*^ mice, we presume that endocochlear potential is not affected, but more studies are needed to confirm this interpretation. Our results also suggest that Gpr125 is not required for the maintenance and function of multiple other cochlear cell types. The lack of phenotype is possibly because of redundant regulatory elements of the PCP pathway or other adhesion GPR molecules. There are likely redundant PCP signals to direct and maintain hair cell orientation, evinced by recent studies on the interaction of Wnt secretion and PCP proteins (Landin Malt et al., 2020; Najarro et al., 2020).

Knockout mice of another adhesion GPR protein, Celsr1, has been shown to display PCP defects, including that of the cochlear and vestibular organs, and abnormal brain development (Curtin et al., 2003; Boutin et al., 2012; Duncan et al., 2017; Obara et al., 2017). Of note, development of type II SGNs, which make a distinctive 90° turn toward the cochlear base to synapse OHCs during cochlear development, was perturbed in *Celsr1*-deficent cochlea (Ghimire et al., 2018). In our study, we do not rule out more subtle defects such as type II SGNs neurite patterning in *Gpr125*^*lacZ/lacZ*^ mice.

The second possible explanation for the lack of phenotype is due to compensation of Gpr125 by other adhesion GPR proteins that are not yet appreciated. Recent studies of Gpr56, Gpr124, and Gpr126 implicate adhesion GPRs in diverse development processes, including brain development, blood vessel formation, and myelination in mammals (Mogha et al., 2013; Chang et al., 2017; Sawal et al., 2018). According to published single-cell transcriptomic data, the embryonic and neonatal cochlear duct express several other adhesion GPRs (e.g., Gpr116, Gpr56, Gpr64, and Gpr126) but not others (e.g., Gpr123, Gpr124, Gpr110, Gpr97) (Kolla et al., 2020). Therefore, adhesion GPR members other than Gpr125 may serve redundant functions.

## Conclusion

In summary, our study reveals that Gpr125 is dynamically expressed in the embryonic and postnatal cochlea. Gpr125 robustly and consistently labels the LER and its derivatives, whose function remains poorly understood. Since Gpr125 is dispensable for cochlear development and function, the *Gpr125^*lacZ*/+^* reporter mice may be useful for cell sorting experiments to further interrogate LER cells, which have been shown to display progenitor cell characteristics (Zhai et al., 2005; Huang et al., 2009). Alternatively, a Gpr125-Cre knock-in mice can be generated for cell-specific manipulation. Thus, the current study may help further our understanding of cochlear development, function, and regeneration.

## Materials and Methods

### Mice

*Gpr125*^*lacZ/+*^ mice were generated by Deltagen (access number XM_1320, San Carlos, CA, United States) and were a kind gift from C.J. Kuo (Stanford University, CA, United States). To determine embryonic age, male *Gpr125*^*lacZ/+*^ mouse was mated with female *Gpr125*^*lacZ/+*^ mouse. The next morning, the vaginal plug was checked. The female was separated if a plug was present, and that noon was designated as embryonic age 0.5. Both male and female mice were examined. Animal care and all experimental procedures were carried out in accordance with institutional guidelines at Stanford University (protocol # 18606).

### Genotyping

Genomic DNA extracted from mouse tails was digested in 1 M NaOH at 98°C for 1 h followed by the addition of 20 μl of 1 M Tris-HCl (pH 8.0). KAPA Taq PCR master mix was used to amplify DNA fragments. The primers used were as follows: *Gpr125* Forward: 5′-GAwAGGCTGTGGGCAGTTGA CAGCAG-3′; *Gpr125* Neo: 5′-GACGAGTTCTTCTGAGGGGA TCGATC-3′; *Gpr125* Reverse: 5′-GCCCGTGACCATTTT TTGTCTCCTC-3′.

### Immunofluorescence Staining

Immunofluorescence was performed as previously described (Atkinson et al., 2018). Whole mount cochleae were isolated and fixed in 4% paraformaldehyde for 40 min (in PBS, pH 7.4; Electron Microscopy Services) at room temperature. P30 otic capsules were decalcified in 500 mM EDTA for 2 days at 4°C. Cochlea from mice of different ages was dissected into three turns, with the Reissner’s membrane, tectorial membrane, and stria vascularis removed. Then, tissues were washed with 0.1% Triton X-100 in PBS (PBST) three times for 5 to 10 min each and blocked with 5% donkey serum, 0.1% Triton X-100, 1% BSA, and 0.02% sodium azide (NaN_3_) in PBS at pH 7.4 for 1 h at room temperature. Next, tissues were incubated with primary antibodies in the same blocking solution overnight at 4°C. The following day, tissues were washed with 0.1% Triton X-100 in PBS and incubated with secondary antibodies diluted in PBS containing 0.1% Triton X-100, 1% BSA, and 0.02% NaN_3_ for 2 h at room temperature. After washing with PBS three times for 10 min, tissues were mounted in Antifade Fluorescence Mounting Medium (Dako, Agilent) and coverslipped.

For sections, cochleae were harvested on ice and fixed in 4% PFA overnight. Then, tissues were sequentially submerged in 10, 20, and 30% sucrose prior to being embedded in 100% OCT and frozen on dry ice. Serial sections were cut at 10 μm with a cryostat. Frozen slides were warmed for 30 min at room temperature and washed in PBS before incubating in PBST for 15 min to permeabilize the tissue. Sections were then treated the same as whole mount tissues.

The following primary antibodies were used: anti-Myosin7a (Rabbit, 1:1000, Proteus Bioscience, 25-6790), anti-Sox2 (Goat, 1:400, R&D, AF2018), anti-CD44 (Rat, 1:200, BD Pharmingen, 550538), anti-β-galactose (Chicken, 1:500, Abcam, ab9361), and anti-Tuj1 (Mouse, 1:500, Neuromics, 801201). Fluorescence-conjugated phalloidin (1:1,000, Invitrogen, Thermo Fisher Scientific, A22283), DAPI (1:10,000, Invitrogen, Thermo Fisher Scientific, D1306), Alexa Fluor donkey anti-goat 647 (1:200, Thermo Fisher Scientific, A21447), Fluor donkey anti-mouse 546 (1:200, Thermo Fisher Scientific, A10036), Alexa Fluor donkey anti-rabbit 546 (1:500, Thermo Fisher Scientific, A10040), Alexa Fluor donkey anti-rabbit 647 (1:200, Thermo Fisher Scientific, A31573), Alexa Fluor donkey anti-chicken 488 (1:500, Thermo Fisher Scientific, A10040), and Alexa Fluor donkey anti-rat 647 secondary antibodies (1:200; Thermo Fisher Scientific) were also used.

### *In situ* Hybridization

*In situ* hybridization was performed as previously described (Jansson et al., 2019). Briefly, tissues were fixed and processed as for immunohistochemistry. The red chromogenic RNAscope kit (Red V2.5 HD, ACDBio, Newark, CA, 322350) was used following the manufacturer’s instructions. Probes used were as follows: Mm-Adgra3-O1 (ACDBio, 827281), which was designed to detect exons 15–19 of *Gpr125* (also known as *Adgra3*), *Dapb* as negative control (ACDBio, 310043), and *Polr2a* as positive control (ACDBio, 312471).

### Auditory Measurements

Auditory brainstem responses (ABRs) and distortion product otoacoustic emission (DPOAE) responses were performed in a sound-isolated and electrically shielded chamber (Atkinson et al., 2018). Mice at P30 ± 2 and P120 ± 2 were anesthetized with a mixture of xylazine (10 mg/kg) and ketamine (100 mg/kg). Body temperature was maintained near 37°C with a heating pad. ABR signals were measured from a needle electrode inserted inferior to the left ear, referenced to an electrode inserted at the vertex of the skull, and a ground electrode was inserted at the hind leg. Tone burst stimuli were delivered at frequencies 4, 5.7, 8, 11.3, 16, 22.6, 32, and 45.3 kHz and sound intensities were raised from 10 to 80 dB sound pressure level (SPL) in 10-dB steps. Up to 512 trials were averaged at each sound level and frequency.

DPOAEs were measured by a probe tip microphone in the external auditory canal. The sound stimuli were two 1-s sine wave tones of differing frequencies (F2 = 1.22 × F1). F2 was varied from 4 to 45.3 kHz, and the intensities of two tones were from 20 to 80 dB SPL with 10-dB steps. The amplitude of the cubic distortion product was measured at 2 × F1-F2. The threshold at each frequency was calculated when the DPOAE was > 5 dB SPL and 2 SDs above the noise level. For statistical analyses of both ABR and DPOAE responses, a lack of response is designated 80 dB SPL.

### Image Analyses, Quantification, and Statistics

Cell quantification and measurements were performed using Fiji ImageJ (NIH). Whole mount preparation or sections of one cochlea from each animal were used for cell counting. The samples were scanned in z-stack mode at 1-μm intervals using confocal microscopy (Zeiss LSM700 confocal microscope, Oberkochen, Germany).

For quantification, Tuj1^+^ SGNs were measured in one to three representative 50 μm^2^ grids for each cochlea. For comparisons of immunofluorescence intensity from *Gpr125 mRNA in situ* hybridization, images were acquired using identical settings for all experimental groups. Immunofluorescence intensity was measured in regions of interest using Fiji ImageJ (NIH). All cell numbers and measurements were presented as mean ± SD. Cell counts, ABR, and DPOAE were compared by a one-way ANOVA (SPSS 20, IBM Armonk, NY). *p* < 0.05 is considered statistically significant.

## Data Availability Statement

The raw data supporting the conclusions of this article will be made available by the authors, without undue reservation.

## Ethics Statement

The animal study was reviewed and approved by the Stanford University.

## Author Contributions

HS and AC conceived and designed the experiments. HS, PA, SB, and WD performed the experiments. HS, TW, SB, and AC analyzed the data. HS, TW, and AC wrote the manuscript. All authors contributed to the article and approved the submitted version.

## Conflict of Interest

The authors declare that the research was conducted in the absence of any commercial or financial relationships that could be construed as a potential conflict of interest.

## Publisher’s Note

All claims expressed in this article are solely those of the authors and do not necessarily represent those of their affiliated organizations, or those of the publisher, the editors and the reviewers. Any product that may be evaluated in this article, or claim that may be made by its manufacturer, is not guaranteed or endorsed by the publisher.

## Supplementary Material

Supplementary Figure 1 Expression of Gpr125-β-Gal in spiral ganglion neurons in E15.5 *Gpr125*^*lacZ/+*^ mice. **(A,A’)** Representative low magnification images of cochlear sections of E15.5 *Gpr125*^*lacZ/+*^ mice immunostained for Gpr125-β-Gal (green) and Tuj1 (red). Gpr125-β-Gal was detected in Tuj1^+^ spiral ganglion neurons (SGN) (dashed line), albeit at lower level than the cochlear duct. **(B–D)** High-magnification images of SGNs from each turn shown in panel **(A)**. There were few β-Gal^+^, Tuj1^+^ cells in the apical turn and many β-Gal^+^, Tuj1^+^ cells (arrowheads) in the middle and basal turns, respectively.

Supplementary Figure 2 Gpr125 is dispensable for cochlear development. **(A)** No significant differences were seen among the lengths of wild-type, *Gpr125*^+/lacZ^ and *Gpr125*^*lacZ/lacZ*^ cochleae. **(B–J”)** Whole mount preparation of wild-type and *Gpr125*^*lacZ/lacZ*^ at E15.5, E18.5, P0, P4 and P30. Immunostaining for Myosin7a, F-actin and Sox2, demonstrated no loss of hair cells, hair bundles, or supporting cells in the *Gpr125*^*lacZ/lacZ*^ cochleae. Images were taken from the middle turn. **(L–N)** Hair cells and supporting cell subtypes were quantified, showing no differences in cell counts among wild-type, *Gpr125*^+/lacZ^ and *Gpr125*^*lacZ/lacZ*^ cochleae (*p* > 0.05, One-way ANOVA). Data are presented as mean ± SD.

## References

[B1] Atkinson P. J. Dong Y. Gu S. Liu W. Najarro E. H. Udagawa T. (2018). Sox2 haploinsufficiency primes regeneration and Wnt responsiveness in the mouse cochlea. *J. Clin. Invest.* 128 1641–1656. 10.1172/jci97248 29553487 PMC5873847

[B2] Aw W. Y. Heck B. W. Joyce B. Devenport D. (2016). Transient tissue-scale deformation coordinates alignment of planar cell polarity junctions in the mammalian skin. *Curr. Biol.* 26 2090–2100. 10.1016/j.cub.2016.06.030 27451904 PMC5005808

[B3] Basch M. L. Brown R. M. II Jen H. I. Groves A. K. (2016). Where hearing starts: the development of the mammalian cochlea. *J. Anat.* 228 233–254. 10.1111/joa.12314 26052920 PMC4718162

[B4] Boucherie C. Boutin C. Jossin Y. Schakman O. Goffinet A. M. Ris L. (2018). Neural progenitor fate decision defects, cortical hypoplasia and behavioral impairment in Celsr1-deficient mice. *Mol. Psychiatry* 23 723–734. 10.1038/mp.2017.236 29257130 PMC5822457

[B5] Bousfiha A. Bakhchane A. Charoute H. Detsouli M. Rouba H. Charif M. (2017). Novel compound heterozygous mutations in the GPR98 (USH2C) gene identified by whole exome sequencing in a Moroccan deaf family. *Mol. Biol. Rep.* 44 429–434. 10.1007/s11033-017-4129-9 28951997

[B6] Boutin C. Goffinet A. M. Tissir F. (2012). Celsr1-3 cadherins in PCP and brain development. *Curr. Top. Dev. Biol.* 101 161–183. 10.1016/b978-0-12-394592-1.00010-7 23140629

[B7] Chang J. Mancuso M. R. Maier C. Liang X. Yuki K. Yang L. (2017). Gpr124 is essential for blood-brain barrier integrity in central nervous system disease. *Nat. Med.* 23 450–460. 28288111 10.1038/nm.4309 PMC5559385

[B8] Chen G. Yang L. Begum S. Xu L. (2010). GPR56 is essential for testis development and male fertility in mice. *Dev. Dyn.* 239 3358–3367. 10.1002/dvdy.22468 20981830 PMC2991479

[B9] Chen P. Johnson J. E. Zoghbi H. Y. Segil N. (2002). The role of Math1 in inner ear development: uncoupling the establishment of the sensory primordium from hair cell fate determination. *Development* 129 2495–2505. 10.1242/dev.129.10.2495 11973280

[B10] Chrysostomou E. Zhou L. Darcy Y. L. Graves K. A. Doetzlhofer A. Cox B. C. (2020). The notch ligand Jagged1 is required for the formation, maintenance, and survival of hensen’s cells in the mouse cochlea. *J. Neurosci.* 40 9401–9413. 10.1523/jneurosci.1192-20.2020 33127852 PMC7724135

[B11] Cullen M. Elzarrad M. K. Seaman S. Zudaire E. Stevens J. Yang M. Y. (2011). GPR124, an orphan G protein-coupled receptor, is required for CNS-specific vascularization and establishment of the blood-brain barrier. *Proc. Natl. Acad. Sci. U. S. A.* 108 5759–5764. 10.1073/pnas.1017192108 21421844 PMC3078373

[B12] Curtin J. A. Quint E. Tsipouri V. Arkell R. M. Cattanach B. Copp A. J. (2003). Mutation of Celsr1 disrupts planar polarity of inner ear hair cells and causes severe neural tube defects in the mouse. *Curr. Biol.* 13 1129–1133. 10.1016/s0960-9822(03)00374-9 12842012

[B13] Doudney K. Stanier P. (2005). Epithelial cell polarity genes are required for neural tube closure. *Am. J. Med. Genet. C Semin. Med. Genet.* 135C 42–47. 10.1002/ajmg.c.30052 15800847

[B14] Driver E. C. Kelley M. W. (2020). Development of the cochlea. *Development* 147:dev162263. 32571852 10.1242/dev.162263 PMC7327997

[B15] Driver E. C. Northrop A. Kelley M. W. (2017). Cell migration, intercalation and growth regulate mammalian cochlear extension. *Development* 144 3766–3776. 28870992 10.1242/dev.151761 PMC5675446

[B16] Duncan J. S. Stoller M. L. Francl A. F. Tissir F. Devenport D. Deans M. R. (2017). Celsr1 coordinates the planar polarity of vestibular hair cells during inner ear development. *Dev. Biol.* 423 126–137. 10.1016/j.ydbio.2017.01.020 28159525 PMC5369242

[B17] Ganesh R. A. Venkataraman K. Sirdeshmukh R. (2020). GPR56: an adhesion GPCR involved in brain development, neurological disorders and cancer. *Brain Res.* 1747:147055. 10.1016/j.brainres.2020.147055 32798453

[B18] Ghimire S. R. Ratzan E. M. Deans M. R. (2018). A non-autonomous function of the core PCP protein VANGL2 directs peripheral axon turning in the developing cochlea. *Development* 145:dev159012. 29784671 10.1242/dev.159012 PMC6031407

[B19] Hamann J. Aust G. Arac D. Engel F. B. Formstone C. Fredriksson R. (2015). International union of basic and clinical pharmacology. XCIV. adhesion G protein-coupled receptors. *Pharmacol. Rev.* 67 338–367. 25713288 10.1124/pr.114.009647 PMC4394687

[B20] Hasson T. Heintzelman M. B. Santos-Sacchi J. Corey D. P. Mooseker M. S. (1995). Expression in cochlea and retina of myosin VIIa, the gene product defective in Usher syndrome type 1B. *Proc. Natl. Acad. Sci. U. S. A.* 92 9815–9819. 10.1073/pnas.92.21.9815 7568224 PMC40893

[B21] Hertzano R. Puligilla C. Chan S. L. Timothy C. Depireux D. A. Ahmed Z. (2010). CD44 is a marker for the outer pillar cells in the early postnatal mouse inner ear. *J. Assoc. Res. Otolaryngol.* 11 407–418. 10.1007/s10162-010-0211-x 20386946 PMC2914240

[B22] Huang Y. Chi F. Han Z. Yang J. Gao W. Li Y. (2009). New ectopic vestibular hair cell-like cells induced by Math1 gene transfer in postnatal rats. *Brain Res.* 1276 31–38. 10.1016/j.brainres.2009.04.036 19397899

[B23] Jansson L. Ebeid M. Shen J. W. Mokhtari T. E. Quiruz L. A. Ornitz D. M. (2019). beta-Catenin is required for radial cell patterning and identity in the developing mouse cochlea. *Proc. Natl. Acad. Sci. U. S. A.* 116 21054–21060. 10.1073/pnas.1910223116 31570588 PMC6800344

[B24] Kolla L. Kelly M. C. Mann Z. F. Anaya-Rocha A. Ellis K. Lemons A. (2020). Characterization of the development of the mouse cochlear epithelium at the single cell level. *Nat. Commun.* 11:2389. 32404924 10.1038/s41467-020-16113-y PMC7221106

[B25] Landin Malt A. Hogan A. K. Smith C. D. Madani M. S. Lu X. (2020). Wnts regulate planar cell polarity via heterotrimeric G protein and PI3K signaling. *J. Cell Biol.* 219:e201912071. 32805026 10.1083/jcb.201912071 PMC7659710

[B26] Li X. Roszko I. Sepich D. S. Ni M. Hamm H. E. Marlow F. L. (2013). Gpr125 modulates dishevelled distribution and planar cell polarity signaling. *Development* 140 3028–3039. 10.1242/dev.094839 23821037 PMC3699285

[B27] McGee J. Goodyear R. J. McMillan D. R. Stauffer E. A. Holt J. R. Locke K. G. (2006). The very large G-protein-coupled receptor VLGR1: a component of the ankle link complex required for the normal development of auditory hair bundles. *J. Neurosci.* 26 6543–6553. 10.1523/jneurosci.0693-06.2006 16775142 PMC2682555

[B28] Mogha A. Benesh A. E. Patra C. Engel F. B. Schoneberg T. Liebscher I. (2013). Gpr126 functions in Schwann cells to control differentiation and myelination via G-protein activation. *J. Neurosci.* 33 17976–17985. 10.1523/jneurosci.1809-13.2013 24227709 PMC3828454

[B29] Monk K. R. Oshima K. Jors S. Heller S. Talbot W. S. (2011). Gpr126 is essential for peripheral nerve development and myelination in mammals. *Development* 138 2673–2680. 10.1242/dev.062224 21613327 PMC3109596

[B30] Morsli H. Choo D. Ryan A. Johnson R. Wu D. K. (1998). Development of the mouse inner ear and origin of its sensory organs. *J. Neurosci.* 18 3327–3335. 10.1523/jneurosci.18-09-03327.1998 9547240 PMC6792659

[B31] Moteki H. Yoshimura H. Azaiez H. Booth K. T. Shearer A. E. Sloan C. M. (2015). USH2 caused by GPR98 mutation diagnosed by massively parallel sequencing in advance of the occurrence of visual symptoms. *Ann. Otol. Rhinol. Laryngol.* 124 Suppl 1 123S–128S. 25743181 10.1177/0003489415574070 PMC4441826

[B32] Munnamalai V. Fekete D. M. (2020). The acquisition of positional information across the radial axis of the cochlea. *Dev. Dyn.* 249 281–297. 10.1002/dvdy.118 31566832 PMC8591551

[B33] Najarro E. H. Huang J. Jacobo A. Quiruz L. A. Grillet N. Cheng A. G. (2020). Dual regulation of planar polarization by secreted Wnts and Vangl2 in the developing mouse cochlea. *Development* 147:dev191981. 32907846 10.1242/dev.191981 PMC7561480

[B34] Obara N. Suzuki Y. Irie K. Shibata S. (2017). Expression of planar cell polarity genes during mouse tooth development. *Arch. Oral Biol.* 83 85–91. 10.1016/j.archoralbio.2017.07.008 28734144

[B35] Peeters R. P. Ng L. Ma M. Forrest D. (2015). The timecourse of apoptotic cell death during postnatal remodeling of the mouse cochlea and its premature onset by triiodothyronine (T3). *Mol. Cell Endocrinol.* 407 1–8. 10.1016/j.mce.2015.02.025 25737207 PMC4390549

[B36] Pickering C. Hagglund M. Szmydynger-Chodobska J. Marques F. Palha J. A. Waller L. (2008). The adhesion GPCR GPR125 is specifically expressed in the choroid plexus and is upregulated following brain injury. *BMC Neurosci.* 9:97. 18834514 10.1186/1471-2202-9-97 PMC2571103

[B37] Sawal H. A. Harripaul R. Mikhailov A. Vleuten K. Naeem F. Nasr T. (2018). Three mutations in the bilateral frontoparietal polymicrogyria gene GPR56 in Pakistani intellectual disability families. *J. Pediatr. Genet.* 7 60–66. 29707406 10.1055/s-0037-1612591 PMC5916802

[B38] Seandel M. James D. Shmelkov S. V. Falciatori I. Kim J. Chavala S. (2007). Generation of functional multipotent adult stem cells from GPR125+ germline progenitors. *Nature* 449 346–350. 10.1038/nature06129 17882221 PMC2935199

[B39] Vizurraga A. Adhikari R. Yeung J. Yu M. Tall G. G. (2020). Mechanisms of adhesion G protein-coupled receptor activation. *J. Biol. Chem.* 295 14065–14083. 10.1074/jbc.rev120.007423 32763969 PMC7549034

[B40] Wu Y. Chen W. Gong L. Ke C. Wang H. Cai Y. (2018). Elevated G-protein receptor 125 (GPR125) expression predicts good outcomes in colorectal cancer and inhibits Wnt/beta-catenin signaling pathway. *Med. Sci. Monit.* 24 6608–6616. 10.12659/msm.910105 30231258 PMC6225730

[B41] Zhai S. Shi L. Wang B. E. Zheng G. Song W. Hu Y. (2005). Isolation and culture of hair cell progenitors from postnatal rat cochleae. *J. Neurobiol.* 65 282–293. 10.1002/neu.20190 16155904

[B42] Zou J. Mathur P. D. Zheng T. Wang Y. Almishaal A. Park A. H. (2015). Individual USH2 proteins make distinct contributions to the ankle link complex during development of the mouse cochlear stereociliary bundle. *Hum. Mol. Genet.* 24 6944–6957. 26401052 10.1093/hmg/ddv398 PMC4654051

