# Supplementary figures and images for: Gpr125 Marks Distinct Cochlear Cell Types and Is Dispensable for Cochlear Development and Hearing
Source: Front Cell Dev Biol. 2021 Jul 28;9:690955. doi: 10.3389/fcell.2021.690955 (PMC8355630; doi:10.3389/fcell.2021.690955)

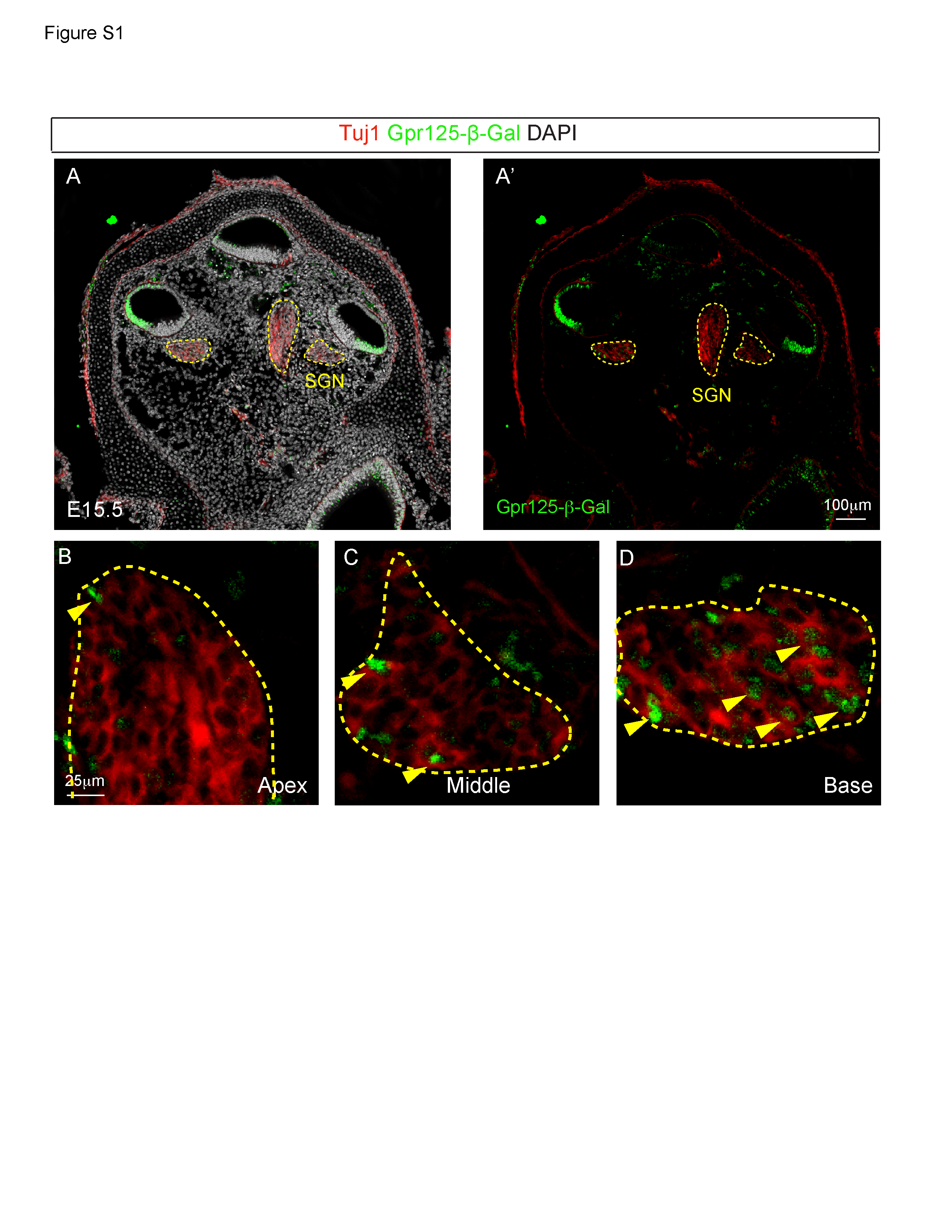

Supplement: Supplementary Figure 1 — Expression of Gpr125-β-Gal in spiral ganglion neurons in E15.5 Gpr125lacZ/+ mice. (A,A’) Representative low magnification images of cochlear sections of E15.5 Gpr125lacZ/+ mice immunostained for Gpr125-β-Gal (green) and Tuj1 (red). Gpr125-β-Gal was detected in Tuj1+ spiral ganglion neurons (SGN) (dashed line), albeit at lower level than the cochlear duct. (B–D) High-magnification images of SGNs from each turn shown in panel (A). There were few β-Gal+, Tuj1+ cells in the apical turn and many β-Gal+, Tuj1+ cells (arrowheads) in the middle and basal turns, respectively. [file Image_1.TIF]

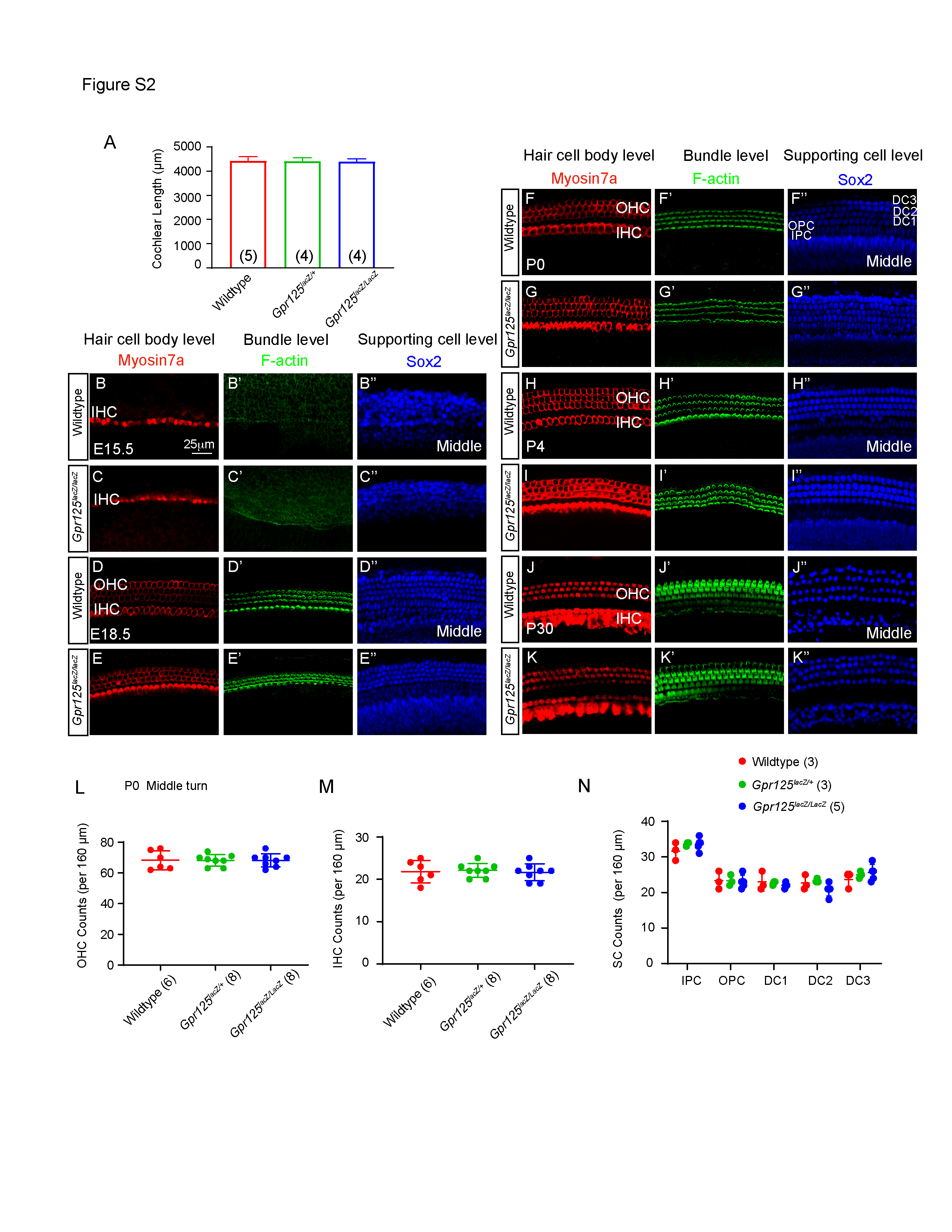

Supplement: Supplementary Figure 2 — Gpr125 is dispensable for cochlear development. (A) No significant differences were seen among the lengths of wild-type, Gpr125+/lacZ and Gpr125lacZ/lacZ cochleae. (B–J”) Whole mount preparation of wild-type and Gpr125lacZ/lacZ at E15.5, E18.5, P0, P4 and P30. Immunostaining for Myosin7a, F-actin and Sox2, demonstrated no loss of hair cells, hair bundles, or supporting cells in the Gpr125lacZ/lacZ cochleae. Images were taken from the middle turn. (L–N) Hair cells and supporting cell subtypes were quantified, showing no differences in cell counts among wild-type, Gpr125+/lacZ and Gpr125lacZ/lacZ cochleae (p > 0.05, One-way ANOVA). Data are presented as mean ± SD. [file Image_2.TIF]
